# Educating religious leaders to promote uptake of male circumcision in Tanzania: a cluster randomised trial

**DOI:** 10.1016/S0140-6736(16)32055-4

**Published:** 2017-03-18

**Authors:** Jennifer A Downs, Agrey H Mwakisole, Alphonce B Chandika, Shibide Lugoba, Rehema Kassim, Evarist Laizer, Kinanga A Magambo, Myung Hee Lee, Samuel E Kalluvya, David J Downs, Daniel W Fitzgerald

**Affiliations:** a Center for Global Health, Department of Medicine, Weill Cornell Medical College, New York, NY, USA; b St Paul College, Mwanza, Tanzania; c Department of Surgery, Bugando Medical Centre, Mwanza, Tanzania; d Care and Treatment Centre, Bugando Medical Centre, Mwanza, Tanzania; e Department of Medicine, Bugando Medical Centre, Mwanza, Tanzania; f Fuller Theological Seminary, Pasadena, California

## Abstract

**Background:**

Male circumcision is being widely deployed as an HIV prevention strategy in countries with high HIV incidence, but its uptake in sub-Saharan Africa has been below targets. We did a study to establish whether educating religious leaders about male circumcision would increase uptake in their village.

**Methods:**

In this cluster randomised trial in northwest Tanzania, eligible villages were paired by proximity (<60 km) and the time that a free male circumcision outreach campaign from the Tanzanian Ministry of Health became available in their village. All villages received the standard male circumcision outreach activities provided by the Ministry of Health. Within the village pairs, villages were randomly assigned by coin toss to receive either additional education for Christian church leaders on scientific, religious, and cultural aspects of male circumcision (intervention group), or standard outreach only (control group). Church leaders or their congregations were not masked to random assignment. The educational intervention consisted of a 1-day seminar co-taught by a Tanzanian pastor and a Tanzanian clinician who worked with the Ministry of Health, and meetings with the study team every 2 weeks thereafter, for the duration of the circumcision campaign. The primary outcome was the proportion of male individuals in a village who were circumcised during the campaign, using an intention-to-treat analysis that included all men in the village. This trial is registered with ClinicalTrials.gov, number NCT 02167776.

**Findings:**

Between June 15, 2014, and Dec 10, 2015, we provided education for church leaders in eight intervention villages and compared the outcomes with those in eight control villages. In the intervention villages, 52·8% (30 889 of 58 536) of men were circumcised compared with 29·5% (25 484 of 86 492) of men in the eight control villages (odds ratio 3·2 [95% CI, 1·4–7·3]; p=0·006).

**Interpretation:**

Education of religious leaders had a substantial effect on uptake of male circumcision, and should be considered as part of male circumcision programmes in other sub-Saharan African countries. This study was conducted in one region in Tanzania; however, we believe that our intervention is generalisable. We equipped church leaders with knowledge and tools, and ultimately each leader established the most culturally-appropriate way to promote male circumcision. Therefore, we think that the process of working through religious leaders can serve as an innovative model to promote healthy behaviour, leading to HIV prevention and other clinically relevant outcomes, in a variety of settings.

**Funding:**

Bill & Melinda Gates Foundation, National Institutes of Health, and the Mulago Foundation.

## Introduction

Evidence in support of male circumcision as an HIV prevention strategy among heterosexual men was the subject of a Cochrane Database systematic review^1^ in 2009. The review by Siegfried and colleagues^1^ cited three major randomised controlled trials, done in South Africa,^2^ Kenya,^3^ and Uganda^4^ between 2002 and 2006. All three trials were stopped early when the group randomised to receive male circumcision was found to have a dramatic reduction in HIV incidence within the first 12–24 months. The systematic review estimated a risk reduction between 38% and 66% over this period. In light of these compelling findings, WHO recommends^5^ widespread implementation of voluntary medical male circumcision for HIV prevention in countries with generalised HIV epidemics and a low prevalence of male circumcision. In 2011, 14 target countries in sub-Saharan Africa were prioritised for scale-up of male circumcision, with a goal of achieving national male circumcision rates of 80%, requiring 20·8 million circumcisions by 2016.^6^ However, uptake has been about 50% lower than predicted, with only 10 million circumcisions done by the end of 2015.^5^

The practice of male circumcision is imbued with religious significance in many cultures, and religious beliefs remain major impediments to the acceptance of male circumcision in many parts of sub-Saharan Africa.^7^, ^8^, ^9^ In our previous work in the Mwanza region of northwest Tanzania, Christian church attenders expressed concern that male circumcision was a Muslim practice and that male circumcision programmes offered by the Tanzanian Government were attempts to convert them.^7^ Other common Christian viewpoints were that male circumcision is a practice only for the sexually promiscuous and that Christians should focus on so-called circumcision of the heart rather than their physical bodies. Because of these beliefs, many Tanzanian Christians questioned whether male circumcision was compatible with their faith.

**Research in context**

**Evidence before this study**

Our clinical experience in Tanzania suggested that a church-based intervention could be an effective means to encourage uptake of male circumcision or other healthy behaviours. To investigate what was known on this topic in sub-Saharan Africa, we searched PubMed, Ovid, and the Cochrane Database of Systematic Reviews for articles published between Jan 1, 2000, and Dec 31, 2015. We initially used the following broad MeSH search terms: “religious beliefs”, “religious ethics”, “religion and medicine”, “health behavior”, “prevention”, “prevention measures”, “Africa south of the Sahara”, “HIV infections/prevention and control”, and “HIV infections/transmission”. Among the articles identified using this strategy, we narrowed our focus using the following additional terms: “faith-based interventions”, “church-based”, “church”, “congregation”, “community-based interventions”, and “circumcision, male”. Among the articles identified by these strategies, we sought randomised controlled trials, cluster randomised trials, and systematic reviews. The searches and article selection were done by two of the study team members, who then presented findings to all coauthors for discussion.

The concept of health behaviour promotion in religious communities has been most broadly explored in church congregations in the USA. Work in the USA has largely focused on African-American and Latino populations, which bear a disproportionate burden of HIV and are also more likely than other groups to report a religious affiliation. Williams and colleagues did a systematic review in 2011 of 11 church-based HIV-focused programmes in the USA. They concluded that key components of successful programmes were providing church leaders and congregations with sufficient knowledge of a topic and ensuring that programmes were concordant with a church's religious doctrine. They cited successful church-based initiatives that presented data to congregations about specific health needs in their neighbourhoods and then worked with congregants to decide together on the nature of their HIV intervention efforts.

By contrast, few studies in sub-Saharan Africa have documented the effect of church-based or faith-based teaching to promote healthy behaviour. A 2013 systematic review by Suthar and colleagues investigated community-based approaches to increase voluntary HIV counselling and testing in Africa and North America. The authors established that community-based approaches including door-to-door testing, school or workplace testing, and mobile test sites were effective in increasing HIV testing. The authors mentioned church-based testing only briefly in reference to a single cost-effectiveness study from South Africa. In 2015, a unique cluster randomised trial by Ezeanolue and colleagues randomised Nigerian churches to receive or not to receive health-themed baby showers that promoted HIV testing for pregnant women attending the church. 92% of pregnant women in the intervention group were HIV tested, compared with 55% of controls. The authors concluded that faith-based organisations and congregational centres, which are widely available and widely accessed in sub-Saharan Africa, offer numerous opportunities for health interventions.

**Added value of this study**

Despite major international efforts to increase male circumcision for HIV prevention in sub-Saharan Africa, uptake has been slower than expected. Qualitative data indicates that a major reason might be the unaddressed religious implications of male circumcision in many devout religious communities. Data from the USA and a single randomised trial from sub-Saharan Africa suggest that promoting healthy behaviour within religious communities might be highly effective. Our cluster randomised trial showed that an educational intervention for religious leaders in Tanzania led to major increases in the uptake of male circumcision. These findings strengthen a growing understanding of the fundamental role that religion can have in promoting healthy behaviour in sub-Saharan Africa.

**Implications of all the available evidence**

Our study's findings, when added to the previous evidence base, show the formative role that religious leaders can play in encouraging healthy behaviour among their congregants. The large majority of people in sub-Saharan Africa are devoutly committed to their religious belief and practice, yet to date very few medical and public health interventions in the region have worked within religious communities. The Nigerian church-based study addressed a different HIV prevention issue and was done more than 4500 km away from our work in Tanzania, yet both studies show the potential for major public health gains when HIV prevention efforts are promoted through culturally-adapted religious community interventions in sub-Saharan Africa.

Churches and other religious organisations exert formative influence on the beliefs and behaviours of a large majority of people in sub-Saharan Africa. Tanzania has two principal religions, Christianity and Islam.^10^ Overall, 93% of the population rate religion as very important in their lives, and 83% attend religious services at least weekly.^10^ Traditional beliefs also remain powerful, with at least 60% of Tanzanians routinely seeking medical care from traditional healers.^11^ Religious leaders are among the most highly respected authorities in the villages.^12^ Therefore, in rural Tanzania and many other areas in which uptake of male circumcision is below target, promoting its use within religious communities is a unique opportunity to augment its acceptance.

We hypothesised that the uptake of male circumcision could be increased if the practice was understood and endorsed by local religious leaders, who would then in turn educate their congregations. Building on findings from our focus group work, we designed a culturally adapted curriculum to educate church leaders of both genders about the religious, cultural, and medical aspects of male circumcision. We then did a community cluster randomised trial to establish whether providing this educational intervention for church leaders could increase the uptake of circumcision among men and boys in Tanzania.

## Methods

### Study design

We did a mixed-methods study that included a community cluster randomised trial, followed by focus-group interviews in participating villages after completion of the trial.^13^ Our trial was done in the rural Mwanza region near Lake Victoria in northwest Tanzania, in conjunction with a voluntary medical male circumcision outreach campaign that was being offered by the Tanzanian Ministry of Health in the northwest of the country. The campaign brings a team of clinicians to undertake free male circumcisions in two to three villages at a time. The campaign routinely provides male circumcision and voluntary HIV counselling and testing to 100–200 men and boys per day and typically remains in a village for 3–6 weeks until the demand for circumcision decreases. In the northwest of Tanzania, the dominant Sukuma tribe does not traditionally circumcise, and Christians constituted more than 95% of the population,^14^ resulting in baseline male circumcision rates of about 20% before the circumcision campaign.^14^, ^15^, ^16^ The Tanzanian policy encourages circumcision in men of all ages. The age group that was targeted by the campaign was 10–35 years because it has the highest incidence of HIV; however, all boys and men aged 10 years or older were eligible for circumcision.

This trial was approved by the institutional review boards of Bugando Medical Centre (CREC/009/2014) and the National Institute for Medical Research (NIMR/HQ/R.8a/Vol. IX/1201), both in Tanzania, and Weill Cornell Medical College, NY, USA (1107011800). According to CONSORT guidelines, village leaders were informed of the study objectives and methods and gave permission for participation, and individuals who participated in focus-group interviews provided written informed consent.^17^, ^18^ Study team members received training in the ethical conduct of research, including confidentiality and how to obtain informed consent.

### Participants

The unit of randomisation in the cluster randomised trial was the village. We worked with the campaign to identify eligible village pairs, which were those that were in the rural Mwanza region of Tanzania, located within 60 km of each other, had clearly defined boundaries, and would be targeted by the male circumcision outreach campaign at the same time. Paired villages were therefore matched by geographical proximity and by the start date of the male circumcision in the village, which were prespecified to be the two most important factors for matching. In these rural regions, poor infrastructure, inadequate transportation, and dirt roads lead to minimal contact between villages. Villages were matched in this way to account for variations in numbers of men seeking circumcision that could be associated with different months of the year, caused by factors such as farming responsibilities during wet versus dry seasons and the effect of an election year in Tanzania.

For focus groups, we enrolled individual church leaders from intervention and control villages. In these villages, we invited one church leader from each of six to eight different churches of varying sizes and denominations to participate. Church leaders provided written informed consent for participation in the focus-group discussions.

### Randomisation and masking

Within the village pairs, villages were randomly assigned by a coin-toss to receive or not to receive the intervention. The coin-toss and assignment were done by one of the study investigators (DJD) who did not participate in enrolment or data collection. Assignments were stored in a sealed opaque envelope away from the study sites. Random allocations were not revealed to members of the male circumcision outreach campaign aside from clinicians who taught at the educational seminars. Because of the nature of the intervention, masking of church leaders or their congregations in the intervention villages was not possible.

### Procedures

Both intervention and control villages received the standard community outreach events to promote male circumcision that are provided by the Tanzanian Ministry of Health during their campaign. The standard outreach includes community meetings, drama, broadcast announcements, and distribution of health information brochures, but these events do not specifically target religious leaders. The outreach activities and services provided by the circumcision outreach team were identical in all villages.

In villages that had been randomised to receive the intervention, Christian church leaders of both sexes and all denominations were invited to attend an educational seminar about male circumcision. The opportunity to participate in the educational seminar was explained to all Christian leaders by the village chairman of all Christian denominations. The chairman asked each church in the village to send at least one male and one female leader.

Seminars lasted for 1 day, and used a curriculum that our team developed in 2012 on the basis of our previous focus-group work.^7^ Seminars were done in Kiswahili (the national language) and co-taught by a Tanzanian pastor and a Tanzanian clinician who worked with the Ministry of Health circumcision outreach campaign. Church leaders were taught medical, historical, religious, tribal, and social aspects of male circumcision and given tools to lead their congregations in the understanding and practice of this issue. Church leaders participated in lively discussions about ways in which they could encourage male circumcision within their congregations.

Seminars were provided immediately before or concurrent with the start of the male circumcision campaign at each site randomised to the intervention. Following the educational seminars, our study team continued to meet every 2 weeks with church leaders, both individually and for group discussions, for the duration of the male circumcision campaign in a given village. Our team provided feedback and answered questions for leaders as they implemented teaching programmes in their churches.

We used paper charts, routinely completed by hand for each male individual seeking circumcision, to document the total number of boys and men presenting for circumcision in each study village. We obtained the total number of male individuals living in each village from 2012 Tanzania census data.^19^ We used this data to calculate the overall proportion of male individuals seeking circumcision during the campaign divided by the total population of men and boys per village.

Members of the circumcision outreach campaign staff recorded demographic information of men seeking circumcision and their stated reasons for seeking circumcision manually. This data was abstracted by members of our study team, unlinked from all personal identifiers. Members of our study team did not have contact with these individuals.

We additionally collected qualitative data following completion of the randomised trial by running focus-group interviews in both intervention and control villages. Focus groups were composed of religious leaders and two study team members, and were split by sex. Discussions, led by two trained study team members of the same sex as the groups, took place in Kiswahili in a private setting using guided discussion questions. The group interviews, each lasting approximately 60 min, were recorded with a digital recorder and transcribed and translated by a professional language service in Mwanza.

### Outcomes

The primary study outcome was the proportion of the total male population in a village that presented for male circumcision at the village health centre during the outreach campaign. The numerator of this proportion is the number of men who sought circumcision in each village, as documented at the circumcision clinic. The denominator is the total male population observed and enumerated in each village in the 2012 national census.^19^ The 2012 census data was chosen as the best possible estimate of the target male population because it would not have been feasible to repeat the census in all male individuals in each village (more than 145 000 men and boys) and to establish baseline circumcision status of each male individual before the intervention.

Secondary outcomes were the proportion of men presenting for circumcision who stated that they wanted to be circumcised as a result of discussions in their churches, with a wife or girlfriend, with a teacher, with a friend, or for their own health. We also used the focus groups to assess the prevailing attitudes that religious leaders expressed about male circumcision in intervention versus control villages.

### Statistical analysis

We predicted a coefficient of variation between communities of 0·25^20^ and that the proportion of males who would present for circumcision would be 65% in intervention villages versus 30% in control villages. We therefore calculated that we needed to randomise 16 villages (eight pairs) to have 80% power to detect this difference at an α level of 0·05.

We fitted a mixed-effects logistic regression model to calculate the odds ratio (OR) for circumcision in the intervention compared with the control group. The analysis was done using the person as the unit of analysis. Village pairs and clustering were taken into account with the following features: (1) we incorporate intercorrelation between participants within the same village using an indicator of the individual village as a random effect; (2) we estimate the effect of the intervention using an indicator of the intervention as a fixed effect; and (3) we adjust for the difference between village pairs using an indicator of the village pair as a fixed effect. These features reflect the nature of the study design and data well.

For the secondary outcomes, we analysed the differences in motivation for circumcision between intervention and control villages, as a village-level analysis only. We used logistic regression to compare the secondary outcomes while accounting for intercorrelation within the village pair. Data analysis was done using Stata IC version 13. This trial was registered with ClinicalTrials.gov, number NCT 02167776.

In our qualitative analysis of the post-trial focus group interviews, translated texts were imported into NVivo, version 10. The aim of this analysis was to do a thematic survey to explore views of focus group participants using interpretative phenomenological analysis.^21^, ^22^ We did a stepwise analysis to establish prevailing themes from transcripts, using an independent and then a collaborative group process with the study team. Finally, we selected representative quotations to illustrate major themes.

### Role of the funding source

The funders had no role in the study design, data collection, data analysis, data interpretation, or writing of the report. The corresponding author had full access to all the data in the study and had final responsibility for the decision to submit for publication.

## Results

**Figure 1:**
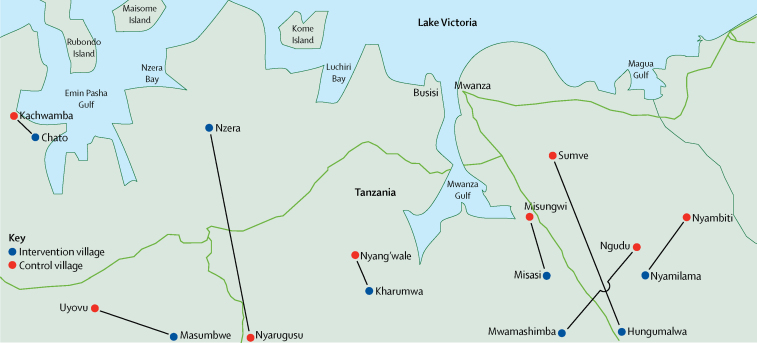
Intervention and control villages selected for the cluster randomised trial in northwest Tanzania Intervention villages, shown in blue, were paired with control villages, shown in red, by proximity (within 60 km) and timing of the male circumcision outreach campaign. Black lines on map indicate paired villages.

**Figure 2:**
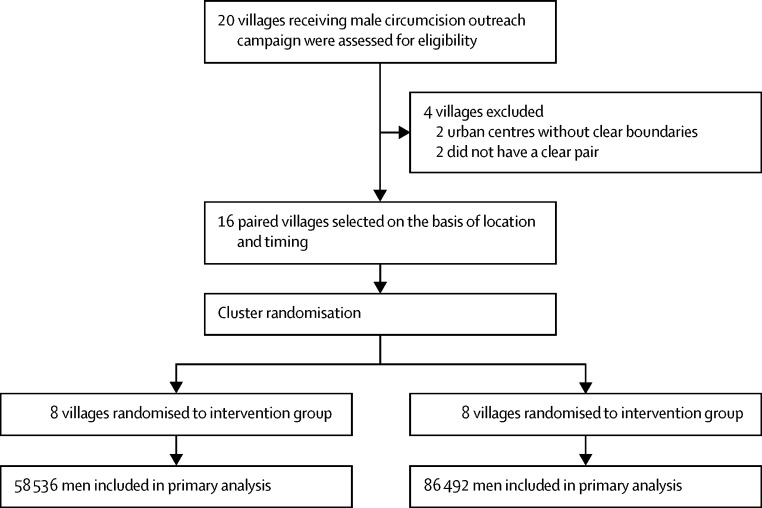
Overview of community randomisation All men in all villages, based on the 2012 Tanzanian census data, were included in the analysis with no individual losses or exclusions.

The first two villages were randomised in this study on June 15, 2014, and data collection was completed on Dec 10, 2015. During this time, we assessed 20 villages in the Mwanza region near Lake Victoria that were scheduled to be recipients of the male circumcision outreach campaign for inclusion in the study. We excluded two of these villages because they were urban centres without clear boundaries, and two others that did not have a clear pair village located within 60 km that would be targeted by the campaign at the same time (figure 1). The remaining 16 villages were paired and subsequently randomised to receive the intervention or control (figure 2). We noted no differences between intervention and control villages in total population, male population, average household size, female-to-male ratio, or educational levels (measured by the national ranking of the village's primary school, determined by test scores; table 1).

In each of the eight intervention villages, the educational seminar was provided to a median of 162 (IQR 138–162) male and female religious leaders. In total, 1194 leaders received training.

**Figure 3:**
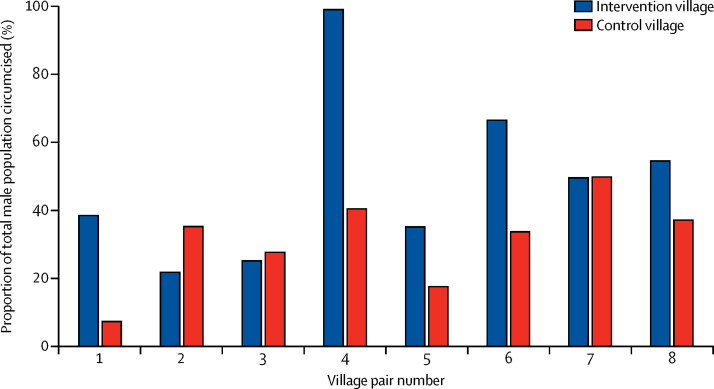
Proportion of total male population circumcised during outreach campaign, by village pairs

In intervention villages in which church leaders received teaching about male circumcision, 52·8% (30 889 of 58 536) of men and boys were circumcised compared with 29·5% (25 484 of 86 492) of men and boys in the eight control villages (OR 3·2 [95% CI 1·4–7·3], p=0·006). The proportion of male individuals seeking circumcision was greater in the intervention group than in the control group in five of the pairs, approximately equal in two of the pairs, and lower than in the control group in one pair (figure 3, table 2). The intracluster correlation coefficient was 0·22 [95% CI 0·08–0·35], which is lower than the predicted value of 0·25 that was used in our sample size calculation. Trials in which clusters have higher intracluster correlation coefficients require more clusters to demonstrate significance; therefore, the intracluster correlation coefficient of 0·22 provided us with higher power than we had predicted.

Demographic data collected from men presenting for circumcision showed no significant differences between the intervention and control village in age or the distribution of religious faiths, classified as Muslim, Christian, or none (table 3). We observed a significant difference between groups in the proportion of men presenting as a result of discussions in church (30·8% in intervention villages *vs* 0·7% in control villages; p<0·0001). Wives and girlfriends in intervention villages were also more likely to have encouraged their male partners to seek circumcision than those in the control villages (p=0·002; table 3).

To investigate the robustness of our findings, we did several sensitivity analyses. First, we removed village pair number 4, which had the most significant difference between invention and control with regard to the number of male individuals who were circumcised. The OR for circumcision comparing intervention and control in the other village pairs remained significant (OR 1·8 [95% CI 1·2–2·9], p=0·01). We did a second sensitivity analysis to account for potential differences in the proportions of Muslims or men who were already circumcised in the villages. Even if 20% more of the population in the control villages than in the intervention villages were circumcised at baseline (which we represented by removing 20% of the denominators in all control villages) our findings remain significant (OR 2·73 [95% CI 1·23–6·04], p=0·013).

After completion of the male circumcision campaign, we ran six focus-group interviews that included 43 church leaders in three intervention and three control villages. Major themes that emerged are presented in the panel. In control villages, church leaders reported numerous rumours that had circulated in their villages about male circumcision and routinely stated that male circumcision was never broached in their churches. Furthermore, most leaders in the control villages felt frustrated and ill-equipped to discuss male circumcision with their congregations. By contrast, church leaders in intervention villages who attended the educational seminars reported a better understanding of male circumcision and a sense of empowerment to teach their congregations about the issue. Many described their congregations as seeking male circumcision in great numbers after church discussions of the topic.

Leaders in both intervention and control villages recognised the strong influence that they had had on the behaviour of their congregation. This sentiment was summarised poignantly by one female leader: “What I ask is that Christian religious leaders should teach a society to uptake male circumcision”.

## Discussion

Our trial shows that equipping and empowering religious leaders to “teach a society to uptake male circumcision” significantly increased the use of voluntary medical male circumcision. We documented an absolute difference of 23·3% with an OR of 3·2 for circumcision in the intervention villages compared with the control villages, with a third of men who were circumcised in intervention villages citing discussions at church as a reason for seeking circumcision. Extrapolating this increase to the country of Tanzania and estimating the variation in baseline rates of circumcision in different regions of Tanzania,^15^, ^19^ our strategy has the potential to lead to more than 1 million additional circumcisions (21·2 million men in mainland Tanzania × 0·29 who are uncircumcised × 0·233 [our percentage increase] =1·44 million men; 95% CI 1·41–1·47 million men), potentially preventing 65 000–200 000 new HIV infections in Tanzania alone.^23^

We believe that a key reason for our study's effectiveness was its attentiveness to structural and cultural factors in promoting behavioural change. A previous community randomised trial to promote adolescent health in Mwanza compared control villages with intervention villages that received a package of condom distribution, adolescent-friendly health services, and reproductive education in schools. The trial showed an increase in adolescents' knowledge, but no effect on their health behaviour or the incidence of sexually-transmitted infections, HIV, or pregnancy.^24^ A process assessment of the trial concluded that a key reason for the absence of effect had been overemphasis on individual cognition with insufficient attention to social and structural determinants of behaviour.^25^ Recognising that most behavioural change in Tanzania operates through a community's social and cultural norms, we designed our trial to work within highly influential religious communities. Furthermore, we grounded our work on the principle that church leaders themselves would be best equipped to establish the most appropriate and effective ways to encourage healthy behaviours among their congregants. For this reason, leaders were given knowledge and tools to teach, but each leader was left to decide how to best address issues of male circumcision in his or her own community.

Our study substantiates the effectiveness of an innovative concept for health promotion in sub-Saharan Africa—namely, drawing on the power of religious leaders to promote healthy behaviour among their congregants. Despite high prevalence of devout religious populations throughout much of Africa,^10^ and some efforts to promote healthy behaviour in church contexts in the USA,^26^, ^27^ few formal assessments of using religion-based interventions to affect health behaviour in Africa have been done. One well designed community randomised trial in Nigerian churches offered specialised baby showers, which included medical teaching and on-site laboratory HIV testing for pregnant women in church congregations.^28^ The Nigerian study documented a major increase in uptake of antenatal HIV tests. Similar to our work in Tanzania, the team in Nigeria concluded that working through highly-accessed religious institutions is an effective way to reach populations even in the most rural communities.

Several of the principles that we have shown to be effective in our trial in Tanzania have been described in reviews of studies from the USA, where the concept of promoting health through religious communities has been more broadly explored than it has in sub-Saharan Africa. A systematic review of 11 church-based programmes that focused on HIV/AIDS in the USA found that providing sufficient information to clergy and ensuring that health messages were congruent with church doctrine were important aspects of successful programmes.^27^ Others have highlighted the importance of designing an intervention that accounts for the cultural context and can be delivered, at least in part, by the community itself.^26^, ^29^, ^30^, ^31^ Our study lays groundwork for additional studies to establish the effect of promoting healthy behaviours through religious communities in sub-Saharan Africa, where the overwhelming majority of the population is deeply committed to religious faith and practice.^10^

The extremely high prevalence of circumcision in one village in our study reflects a unique situation that would be difficult to duplicate. Our study team reported a robust theological debate about male circumcision among church leaders in that particular village. In particular, one highly influential leader preached strongly against male circumcision, insisting that it was a campaign led by foreign devil worshippers who were stealing foreskins for profit. The dissenting leader's views caught the attention of village civic leaders, leading to additional discussions throughout the entire village. Ultimately, the dissenting leader was invited to observe the operations of the male circumcision campaign at the health centre, including witnessing the burning of foreskins on site. The leader changed position and then preached strongly and repeatedly in favour of male circumcision for the remainder of the campaign. We suspect that this debate, which did not occur at other intervention sites, could have further impassioned church leaders who agreed with promoting male circumcision when they discussed these issues with their congregations. This case study suggests that involving not only church but also civic leaders might further increase the effect of our educational intervention.

The sensitivity analysis suggests that our findings remain robust despite the limitations of our study. Because the collection of baseline data on circumcision status and religion from more than 145 000 men in 16 rural villages would not have been feasible, we did our study in a region of Tanzania in which baseline rates of male circumcision were low and more than 95% of the population is Christian. We therefore relied on 2012 national census data, and assumed that paired nearby villages would have similar baseline characteristics. We further assumed that most village residents were not aware of the village's assignment as an intervention or control group because of the breadth of educational activities, outside of the church, that were provided in all study villages. A major strength of our cluster randomised trial is that randomisation should address these and other potential confounders.

In conclusion, we have shown that an intervention to educate church leaders about HIV prevention and male circumcision led to a major increase in uptake of male circumcision. We believe that this innovative concept of inciting behavioural change by working with influential religious leaders should be incorporated as an integral part of all circumcision campaigns. Because of the contextual flexibility of our intervention, we believe that it will be broadly applicable for addressing other health behaviours and for working in other regions and among other religious groups. Moreover, our work has major implications for health-care delivery in much of sub-Saharan Africa, where the large majority of people are highly committed to their faith traditions. We are now adapting this curriculum to educate Muslim leaders and to address other challenging health behaviours in Tanzania.

## Figures and Tables

**Table 1:** Characteristics of study villages

	**Intervention villages (n=8)**	**Control villages (n=8)**
Total population per village*	16 541 (11 451–18 112)	22 033 (11 543–30 319)
Number of male individuals per village*	7936 (5574–8829)	10 728 (5656–14 814)
Household size*	5·9 (5·8–6·3)	5·9 (5·5–6·7)
Sex ratio (number of males per 100 females)*	96·5 (94·5–99·5)	94·5 (92–97·5)
National rank of village primary school (based on test performance)†	4586 (2010–5110)	4151 (860–8358)

Data are median (IQR).

* From United Republic of Tanzania 2012 Population and Housing Census.^19^

† From National Examinations Council of Tanzania (2014 data).

**Table 2:** Differences between number of male individuals circumcised in each village pair

	**Intervention village**			**Control village**			**Percentage increase from control to intervention (95% CI)**
	Village name	Total male population, n	Number of male individuals circumcised	Village name	Total male population, n	Number of males circumcised	
1	Mwamashimba	4601	1777 (38·6%)	Ngudu	13 471	1001 (7·4%)	31·2 (29·7 to 32·7)
2	Hungumalwa	7931	1733 (21·9%)	Sumve	7984	2829 (35·4%)	−13·5 (−14·9 to −12·1)
3	Nyamilama	3724	940 (25·2%)	Nyambiti	6711	1869 (27·8%)	−2·6 (−4·4 to −0·8)
4	Nzera	10 135	10 045 (99·1%)	Nyarugusu	20 567	8350 (40·6%)	58·5 (57·8 to 59·2)
5	Misasi	7941	2794 (35·2%)	Misungwi	14 864	2617 (17·6%)	17·6 (16·4 to 18·8)
6	Kharumwa	6546	4359 (66·6%)	Nyang'wale	4600	1554 (33·8%)	32·8 (31·0 to 34·6)
7	Chato	8343	4148 (49·7%)	Kachwamba	3532	1764 (49·9%)	–0·2 (–2·2 to 1·8)
8	Masumbwe	9315	5093 (54·7%)	Uyovu	14 763	5500 (37·3%)	17·4 (16·1 to 18·7)

**Table 3:** Differences in male individuals who were circumcised in intervention and control villages, according to demographic characteristics and reasons for seeking circumcision

		**Intervention villages (n=30 889)**	Control villages (n=19 984*)	**p value**
Age (years)		15 (12–18)	15 (12–19)	0·8
	>25 years	2187 (7·1%)	1341 (6·8%)	0·44
Marital status				
	Married	7588 (24·6%)	6522 (32·6%)	0·001
	Single	2705 (8·8%)	1721 (8·6%)	0·67
	Minor living with parents	19 049 (61·7%)	11 688 (58·5%)	0·052
Religion				
	Muslim	828 (2·7%)	1060 (5·3%)	0·85
	Christian	25 619 (82·9%)	16 196 (81·0%)	0·76
	None	2974 (9·6%)	2700 (13·5%)	0·94
Reasons for seeking circumcision				
	To promote own health	360 (1·2%)	457 (2·3%)	0·56
	Heard about it in church	9527 (30·8%)	132 (0·7%)	<0·0001
	Heard about it from a friend	5937 (19·2%)	2971 (14·9%)	0·49
	Heard about it from a teacher	3792 (12·3%)	1345 (6·7%)	0·41
	Heard about it from girlfriend or wife	556 (1·8%)	74 (0·4%)	0·002

Data are median (IQR) or number (%), unless otherwise stated.

* Demographic data from one control village was lost.

## Panel Summary of themes and quotes from focus groups done after the conclusion of the male circumcision campaign in intervention and control villages

**Church leaders and church members in control villages were misinformed and suspicious about male circumcision**

• “ ‘Those who have undergone circumcision have gone to have their bodies used for business’—that is now what many people say and they fear to go for circumcision” (male leader, control village)
• “Christians as Christians…they have ideas that are different from Islam. That means they think as soon as they are circumcised it means that one way or another they agree with Islamic thinking” (male leader, control village)

**Church leaders in control villages desired additional teaching about male circumcision**

• “[Those working on the male circumcision campaign] don't bring any seminar saying we come because of this and this and this … they just come all of a sudden coming to do this matter. You would think everyone in this world is educated like they are” (male leader, control village).
• “We answer by guessing. Even in the churches we don't understand the meaning of circumcision. If there would come education in the church we would get the ability to educate someone so that he can do it” (male leader, control village)
• “For me, what I see, religion is not concerned with male circumcision because from the beginning up to my age I did not hear talking about male circumcision in churches” (female leader, control village)

**Church leaders from intervention villages felt empowered and reported high levels of acceptance**

• “We came to see that boy, it [male circumcision] is very important…the way we motivate in the church people received it…Boys, youth, men received it as being meaningful. In the past it had no meaning for us Christians. We saw it as improper that, I don't know, it was shameful. If you preach about circumcision in church you feel like you are preaching insults but gosh, it is something meaningful” (female leader, intervention village)
• “After this seminar of circumcision we delivered the message in our churches. We told people about the children to take them to the health centre to be circumcised. People agreed to this matter…in great numbers. Yes, they did not refuse, they agreed completely” (female leader, intervention village)

**Church leaders from intervention and control villages agreed that working through churches is effective**

• “For those who refused to do circumcision, for Christians the first reason was that people did not have enough information concerning male circumcision in the church… But if people will be taught in the church they will not refuse to take circumcision” (female leader, control village)
• “Pastors have a power to motivate people and church members are trusting their pastors more than any other person or leader. If the pastor will encourage people in church many young men will uptake circumcision” (female leader, control village)
• “What I ask is that Christian religious leaders should teach a society to uptake male circumcision” (female leader, intervention village)
